# Invasive papillary carcinoma of the breast

**DOI:** 10.3389/fonc.2024.1374091

**Published:** 2024-03-27

**Authors:** Shijing Wang, Qingfu Zhang, Xiaoyun Mao

**Affiliations:** ^1^ Department of Breast Surgery, The First Affiliated Hospital of China Medical University, Shenyang, Liaoning, China; ^2^ Department of Pathology, The First Affiliated Hospital of China Medical University, Shenyang, Liaoning, China

**Keywords:** invasive papillary carcinoma, papillary neoplasms, rare breast cancer, pathology, treatment

## Abstract

Invasive papillary carcinoma is a rare form of breast cancer that is more likely to occur in postmenopausal women. Previous studies have been limited to case reports and small retrospective studies, leading to low awareness of this type of tumor and difficult clinical management. According to the available literature, invasive papillary carcinoma exhibits unique pathological features and biological behaviors. Invasive papillary carcinoma is mostly luminal type, with a low rate of lymph node metastasis, which underlies its favorable prognosis. The effectiveness of adjuvant therapy in reducing tumor burden and improving prognosis in patients with invasive papillary carcinoma remains uncertain. Due to the rarity of the lesion, conducting prospective clinical trials is impractical. The use of biological models, such as organoids, can help alleviate the impact of the scarcity of this condition on research. In addition, invasive papillary carcinoma is affected by specific genomic events, and more extensive studies of gene expression profiling may provide molecular-level insights to make optimal therapeutic decisions.

## Introduction

Papillary neoplasms of the breast comprise a broad range of proliferative diseases, including non-cancerous and abnormal growths, as well as malignant lesions. They constitute less than 3% of all breast lesions (1, 2). According to the latest WHO classification of breast tumors (5th edition), papillary neoplasms of the breast include benign and malignant lesions (**Table 1**) (3). Benign papillary neoplasms, also known as intraductal papilloma, are most commonly found in multiparous women and have a low risk of becoming malignant (4). It is important to note that while these benign papillary neoplasms are limited to the ducts of the breast, they do increase a woman’s risk of developing breast cancer. The risk is higher for those with multiple papillomas and about 1.5 to 2.0 times higher for those with solitary papillomas (5, 6). Malignant papillary neoplasms comprise papillary ductal carcinoma *in situ* (DCIS), encapsulated papillary carcinoma (EPC), solid papillary carcinoma (SPC), and invasive papillary carcinoma (IPC) (7, 8). IPC is the most rare malignant papillary neoplasm, accounting for approximately 13-20% of papillary neoplasms and 0.5% of all invasive breast cancer cases (9, 10). In the past, IPC referred to intraductal papillary carcinoma with invasion. The invasive component is usually non-specific types of breast cancer that lack a papillary structure. However, in the 2012 WHO classification of breast tumors (4th edition), IPC is defined as invasive adenocarcinoma with more than 90% papillary structures in the invasive component (11). The definition was unchanged in the 2019 Fifth Edition of the Classification (12). These modifications have resulted in a reduction of available data from studies related to IPC, which has made clinical management more challenging. Therefore, a search was conducted on PubMed using the keywords ‘Invasive papillary carcinoma, Papillary neoplasms, Rare breast cancer, Pathology, Treatment’ to find relevant literature. The inclusion criteria for the search were studies on the clinical presentation, pathology, treatment, and prognosis of IPC, as well as studies on papillary neoplasms. Exclusion criteria were studies that contained the above keywords but were not directly related to the topic. Ninety-eight pieces of literature were included based on the inclusion and exclusion criteria. The literature on IPC includes clinical trials, observational studies, case reports, and review articles. Our aim is to provide a comprehensive overview of research progress in the diagnosis, treatment, and prognosis of IPC from multiple perspectives.

**Table 1:** Papillary neoplasms in the WHO Classification of Breast Tumors (5th edition).

Papillary neoplasms	ICD coding
Intraductal papilloma	8503/0
Ductal carcinoma in situ, papillary	8503/2
Encapsulated papillary carcinoma	8504/2
Encapsulated papillary carcinoma with invasion	8504/3
Solid papillary carcinoma in situ	8509/2
Solid papillary carcinoma with invasion	8509/3
Intraductal papillary adenocarcinoma with invasion	8503/3

## Clinical manifestations

The incidence of IPC is relatively low, accounting for less than 1-2% of newly diagnosed cases of invasive breast cancer (13–15). Interestingly, IPC is the most common type of rare breast cancer in men, accounting for about 2-4% of all cases, due to the less developed terminal ductal lobular unit and larger ducts in men (16, 17). IPC rarely occurs in isolation but is often associated with invasive breast cancer of a non-specific type or papillary DCIS (18). Kline and Kannan reported that the incidence of pure IPC was less than 0.3% (19). Talu et al. conducted a comprehensive review of 1153 cases of invasive breast cancer, of which only 7 cases were pure IPC (0.6%) and 15 cases were mixed IPC (1.3%). Among the mixed IPC cases, other associated histological types of invasive breast cancer included invasive ductal carcinoma (IDC) (100%), invasive micropapillary carcinoma (20%), and pleomorphic lobular carcinoma (6.7%) (20). The co-existence of IPC with other types of breast cancer may increase the likelihood that other invasive cancers will be missed due to the small sample of tissue examined by core needle biopsy (CNB) and its fragmented structure, resulting in an underestimation of the patient’s total lesions (21). In addition, it is important to note that tissue fragility may increase the risk of epithelial displacement in the breast caused by the puncture (22). Although the biological significance of epithelial displacement is unclear, it is important to recognize this processing artefact to avoid misdiagnosis of mesenchymal or lymphoid infiltration. Therefore, complete excision of the suspicious lesion and thorough immunohistochemistry (IHC) is a reliable method for diagnosing IPC.

Unlike benign papillary neoplasms, IPC typically affects postmenopausal women over the age of 50, with a higher incidence in non-Caucasian women (7, 23, 24). However, there is no clear evidence of hereditary factors. Hashmi et al. conducted a study which found that the mean age of IPC patients was 58.77 ± 8.38 years, with the majority being over 50 years of age (68.2%) (25). The study by Zheng et al. showed that 82.4% of patients diagnosed with IPC were over 50 years of age, a significantly higher percentage than the 70.5% of patients with IDC (26). Papillary neoplasms, particularly in women aged 70 years and older, are highly likely to be diagnosed as IPC (10, 27).

Patients with IPC often present with a palpable breast mass and nipple discharge. Bloody nipple discharge occurs in approximately one third of patients due to IPC involving the ductal system of the breast. Tumors are usually located below the nipple and multiple tumor foci are present in 23% of patients (28). In addition, typical IPC are solid or cystic-solid lesions, with texture and volume depending on the cyst-to-solid ratio. In cases where there is a cystic component, the tumor size tends to be larger (29). Huang et al. analyzed 1,147 patients with IPC and 307,279 patients with IDC. The study found that IPC had a higher percentage of tumors greater than 5 cm in diameter compared to IDC (12.3% vs. 7.0%) (30). Hashmi et al. reported that the majority of IPC had sizes between 2-5 cm (71.1%), while a smaller percentage had sizes greater than 5 cm (21.1%) (10). Zheng et al. showed that IPC had a smaller tumor size than IDC, but the proportion of tumors larger than 5 cm was slightly higher (7.1% vs. 5.1%) (26). Histopathologically, lesions should be assessed by the size of solid invasive foci to avoid over-assessment by cystic components. On imaging, IPC typically presents in a variety of forms and lacks specificity. Breast ultrasound typically reveals IPC as a hypoechoic solid mass or a complex cyst with compartmentalization (31, 32). Anechoic areas within these tumors may indicate the presence of cystic components or hemorrhage. Doppler imaging can detect rich blood flow signals in solid components (33). On mammography, the lesion may be seen as an oval or microfollicular dense shadow, which may be surrounded by microcalcifications (34, 35). In addition, malignant papillary neoplasms on MRI with non-mass enhancement tend to show internal clustered ring enhancement, in contrast to benign papillary neoplasms (36).

## Pathologic characteristics

IPC usually originates from epithelial cells in the juxtaductal part of the larger ducts, either in the areola or in the nipple ducts. Although less common, IPC may develop from smaller or medium-sized ducts (37). The gross appearance of IPC depends largely on the proportions of the different components of the tumor and reactive changes in the tissue. The solid component of IPC shows multiple papillary projections with a smooth surface and a tougher texture when stroma fibrosis is severe. Compared with benign papillary neoplasms, IPC appears to be more friable, possibly due to the homogeneous epithelium rather than the normal heterogeneous composition (38). Despite its distinct boundary, it lacks a thick fibrous envelope, similar to that of EPC (39). Additionally, lesions may undergo secondary changes such as inflammation, hyperplasia, metaplasia, and necrosis, resulting in atypical papillary structures (7, 40). It is important to note these changes at the time of diagnosis. Ischemic infarction can occur due to torsion of the apex of the papillary structure or its branches. Ischemic infarction involves the entire papilla, including the glandular epithelium, myoepithelium, and fibrovascular core, while neoplastic necrosis only involves the glandular epithelium. Hemorrhage can occur due to the abundant blood supply to the parenchymal portion of the tumor, either from ischemic infarction or CNB, especially in lesions with fibrosis or previous unhealed hemorrhage (41). Hemorrhage may result in a dark brown appearance, with dark brown blood clots present in most cyst walls and lumens, and solid areas appearing tan or gray.

**Figure 1:**
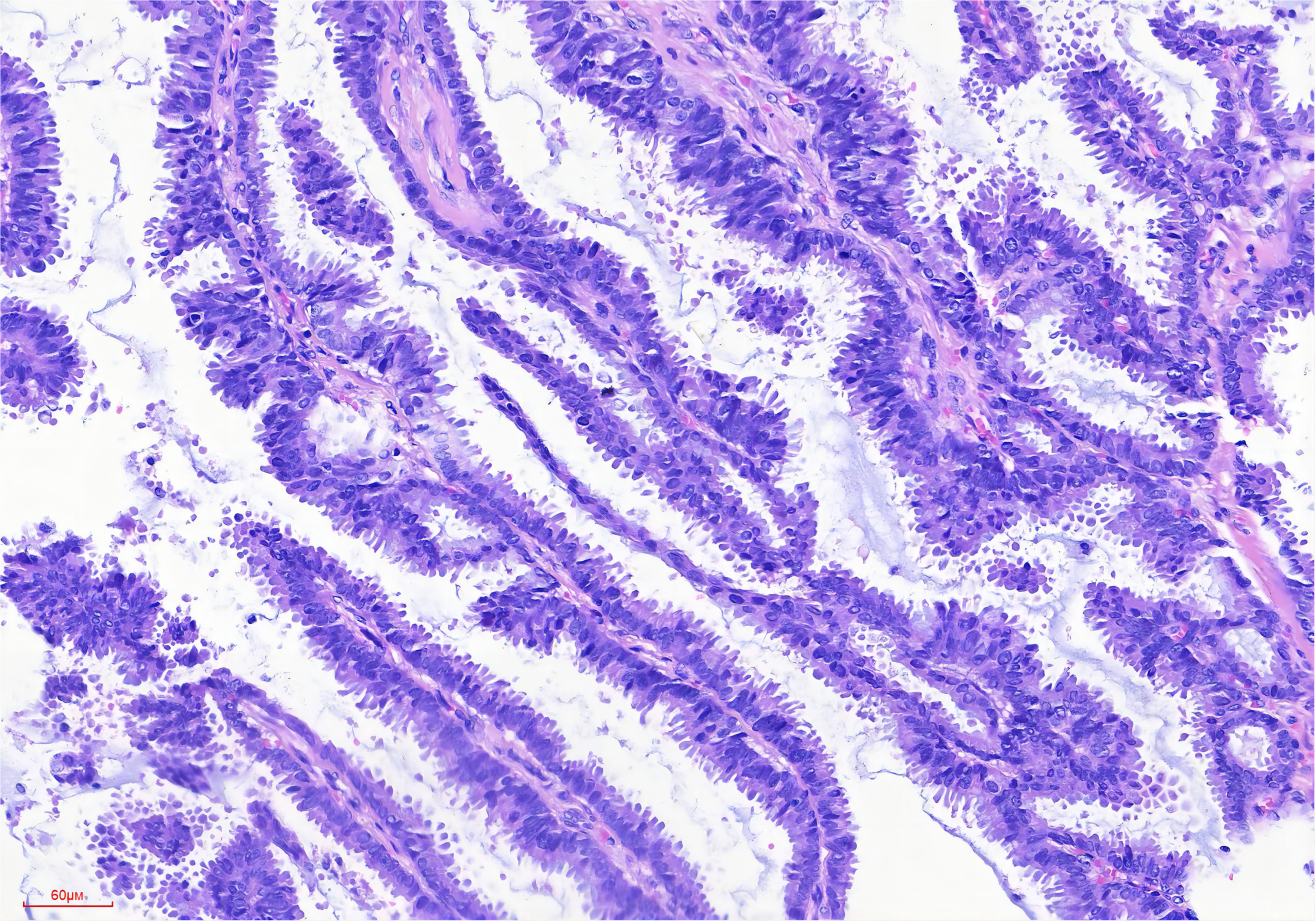
Broad papillary structure with a central fibrovascular core (HE stain, ×200 original magnification).

Microscopically, the IPC exhibits an infiltrative growth pattern, with over 90% of the invasive component comprising papillary structures (42, 43). These structures consist of a fibrovascular core covered with hyperplastic luminal epithelium (**Figure 1**). In comparison, SPC has a denser structure with a narrower fibrovascular core (44). The epithelial cells appear crowded and have light cytoplasmic staining, with a few visible mitotic. According to the Nottingham Histology classification, most of the cells are classified as grade 2 (20). It is worth noting that they may be accompanied by either apocrine metaplasia or apocrine secretion. Most breast cancers with papillary structures are typically characterized as estrogen receptor (ER) positive and human epidermal growth factor receptor 2 (HER2) negative, except for tall cell carcinoma with reversed polarity and mucinous cystadenocarcinoma (45, 46). Typical IPC is characterized by high expression of ER and progesterone receptor (PR), no amplification of the HER2 gene, and a low Ki-67 proliferation index (30, 47). In a study by Hashmi et al., which reviewed 44 patients with IPC, the mean Ki-67 index was found to be 19.95 ± 21.12%. The Ki-67 index was less than 15% in 59.1% of the patients. In 72.7% of cases, ER and PR were expressed, while 86.4% were HER2 negative (25). Furthermore, Talu et al. conducted a study which revealed that 72.7% of the molecular subtypes of IPC were Luminal B, 22.7% were triple negative, and 4.6% were Luminal A (20).

Owing to the rarity of IPC, other metastatic carcinomas featuring papillary structures, such as ovarian serous carcinoma, papillary thyroid carcinoma, and papillary adenocarcinoma of the lung, must first be ruled out as potential diagnoses. Typically, most metastatic cancers exhibit positive results for biomarkers specific to their origin, including, but not limited to, WT-1, PAX-8, TG, TTF-1, and napsin A (48–50). It is important to avoid misclassifying invasive micropapillary carcinoma as IPC, as it may resemble micropapillary structures in the vasculature-like lumen but without a vascular core (51). Additionally, invasive carcinoma that presents in the context of EPC or SPC should not be classified as IPC.

In breast pathology, the classification and evaluation of papillary neoplasms has been one of the most challenging tasks. The reason for this complexity is that the concept of papillary neoplasms is based on morphology, and different types of papillary neoplasms have very similar papillary structure (38). It is a well-established fact that the formation of papillary structures is a dynamic process that is strongly linked to genetic changes and phenotypic characteristics (52–55). Papillary neoplasms often experience chromosomal alterations, including loss of heterozygosity at 16p13 and 16q21, as well as alterations in chromosomes 3, 7, 17, and X (56, 57). Specifically, mutations in chromosome 16 have been found to promote the early onset of papillary neoplasms in the breast. The malignant transformation of papillary neoplasms is significantly associated with *tp53* deletion and loss of heterozygosity at 16q23, which is labeled by D16S476 (58). Roughly two-thirds of benign papillary neoplasms are impacted by mutations in *pik3ca* and *akt* (59). Studies in mouse models of conditional breast cancer have revealed that several genetic pathways, including ERBB, RAS, WNT, CDK2, and LKB1, can induce tumors with a papillary structure (60–62). In some papillary neoplasms that are limited to the ducts, the growth of epithelial cells may affect the connective tissue of the duct wall, resulting in collaborative growth and the formation of papillary structures (63). Furthermore, these papillary structures are not static and may be partially lost during tumor infiltration, leading to the emergence of other forms of invasive breast cancer, such as mucinous carcinoma or invasive micropapillary carcinoma (64, 65). Secondary changes, such as tumor necrosis and interstitial fibrosis, can give rise to pseudopapillary structures (66, 67). Rapidly proliferating invasive breast cancers may undergo ischemic necrosis, which can cause cystic areas to form around blood vessels. These areas may contain residual live tumor cells, and the necrotic pattern with blood vessels and surrounding tumor cells can simulate a papillary structure.

To differentiate between different types of papillary neoplasms, it is crucial to determine if the tumor is benign or malignant and if it is invasive. In 1962, Kraus et al. proposed key features to differentiate between benign and malignant papillary neoplasms (68). These characteristics include epithelial cell types, glandular characteristics, and the relationship between the mesenchyma and epithelium, and have stood the test of time and remain valid today. In addition, certain features have been found to be independently associated with malignancies in papillary neoplasms. In 2015, Loh et al. discovered that cyclin B1 could be used to detect malignant papillary neoplasms with 80% sensitivity and 72.7% specificity through IHC. However, cyclin D1 was found to be less precise with 86.4% sensitivity and 32.6% specificity (69). In 2021, Jamidi et al. conducted a review of the cytology of 153 cases of papillary neoplasms. They found that the absence of myoepithelial cells within papillary structures, the presence of cytoplasmic granules, an increased amount of cytoplasm, and a nuclear/cytoplasmic ratio greater than 0.7 can be used as reference indicators of malignancy. The sensitivity for predicting malignancy with any of these four parameters was 95.1%, and the specificity was 100% (70). Loss of myoepithelial cells is generally considered an indication of tumor infiltration. However, this rule does not entirely apply to papillary neoplasms (**Table 2**) (38). In the case of papillary neoplasms, determining infiltration requires equal consideration of the morphology of the margins of the lesion and the status of the myoepithelial cells. While the absence or reduction of myoepithelial cells is a recognized criterion for IPC the presence of myoepithelial cells does not necessarily exclude a diagnosis if the lesion has a homogeneous cluster of epithelial cells, moderate or high nuclear grade, or an elongated fibrovascular core (71). The possibility of infiltration should be considered if the cancerous tissue has a nested morphology, map-like irregular or toothed margins, and a profibrotic response in the interstitium (1). The outermost layer of EPC is a thick fibrous envelope that lacks myoepithelial cells around it, but because of its slow growth and good prognosis, most of them are considered equivalent to carcinoma in situ. It is important to note that fibrosis at the periphery of papillary neoplasms often involves the glands, leading to a misdiagnosis of invasion.

**Table 2:** The value of myoepithelium in differentiating papillary neoplasms.

Papillary neoplasms	Myoepithelium within papillary structures	Myoepithelium around the duct	Clinical significance
Intraductal papilloma	Positive	Positive	Benign
Ductal carcinoma in situ, papillary	Negative	Positive	Low risk of invasion
Encapsulated papillary carcinoma	Negative	Negative	Equivalent to DCIS
Encapsulated papillary carcinoma with invasion	Negative	Frequently Negative (>85%)	Equivalent to DCIS with invasion
Solid papillary carcinoma in situ	Negative	Positive/Negative	Equivalent to DCIS
Solid papillary carcinoma with invasion	Negative	Usually Negative (>70%)	Equivalent to DCIS with invasion
Intraductal papillary adenocarcinoma with invasion	Negative	Negative	Invasive carcinoma

Pathological diagnosis has traditionally relied on morphology as a benchmark, which is subjective. However, the precision and reliability of diagnosing complex pathologies have significantly improved with the emergence of IHC. According to the recommendations of the WHO working group, myoepithelial cells are evaluated using two to three markers, typically a combination of nuclear and cytoplasmic markers, to increase both sensitivity and specificity (71). P63 is a nuclear marker used to detect myoepithelial cells. It is more sensitive and specific for the myoepithelium, which is not expressed by vascular endothelium or myofibroblasts. Both CK14 and CK5 demonstrate the presence of myoepithelial cells and show varying levels of positivity in the hyperplastic epithelium of benign papillary neoplasms (72). However, staining is reduced in intraductal papillary carcinomas. Furthermore, myoepithelial cells can be identified through the use of cytoplasmic staining markers such as SMMHC, SMA, calponin, and CD10 (73). However, it is important to note that these markers may result in inconsistent sensitivity and specificity, and may also stain perivascular cells in myofibroblasts and the fibrovascular core of papillae, which can impact interpretation (48).

## Biological behaviors

Biological behaviors such as cellular proliferative activity, invasion and metastasis play a critical role in both tumor initiation and progression, as well as patient prognosis. Biological behaviors are known to be genetically determined, and these genetic features can be reflected to some extent by tumor expression subtypes, grading, and staging (74, 75). Currently, the top three vital measures for evaluating the biological behaviors of breast cancer are ER, PR, and HER2 expression by IHC/FISH, grading, and staging of tumors (58). With advancements in molecular biology techniques, our understanding of breast cancer has advanced to the molecular level, revealing the underlying mechanisms of certain biological behaviors.

The IPC has a slow biological activity, tends to progress gradually and has low rates of lymph node and distant organ metastasis. In the current literature, the majority of IPC are classified as luminal type, histologically graded as grade 2 according to the Nottingham system, and have a low TNM stage (3, 24). Hashmi et al. retrospectively compared 44 patients with IPC and 1268 patients with IDC and found that IPC had more favorable pathological features than IDC in terms of tumor T stage, axillary lymph node metastasis, Ki-67 index, PR and HER2 expression (25). Notably, IPC demonstrated higher levels of hormone receptor (HR) positivity, lower mean Ki-67 index, and decreased rates of HER2 amplification than that of IDC. Furthermore, IPC cases exhibited a significantly lower rate of axillary lymph node metastasis (13.6%) than that of IDC cases (50.2%). Additionally tumors with larger size and lymphovascular invasion were less prevalent in IPC compared to that of IDC. Several studies have shown that patients with IPC have a significantly lower rate of axillary lymph node metastasis, ranging from 11.6% to 17.25%, compared to that in invasive breast cancer of a non-specific type, where the rate ranges from 32.6% to 49% (18, 26). In 2017, Suh et al. reported a rare observation in a 59-year-old patient with IPC, who exhibited a 10-year natural history of the disease (76). Despite the primary tumor’s substantial dimensions (10.4 cm x 7.2 cm x 3.5 cm), it had grown less than 2 cm over a decade without treatment, and no distant metastases had emerged. The IHC findings did not show any alterations. The tumor tested positive for ER and PR, but negative for C-erbB2 expression. The Ki-67 labeling index was approximately 10%.

Recent studies have shown that the development and progression of breast cancer is a complex, multi-step process with a genetic basis (77–80). The transformation of cells from a normal to a breast cancer phenotype can be attributed to DNA damage. Similarly, genetic alterations that accumulate over time cause the progression of breast cancer from early to later stages (81). Papillary carcinomas (PC) comprises three histological subtypes: EPC, SPC, and IPC (40, 42). Notably, the genomic expression patterns in IPC, EPC and SPC were highly similar. This similarity provides important clues for further study and understanding of the biological behaviors of PC. PC often shows loss of 16q and gain of 16p and 1q, which is consistent with the genomic alteration pattern observed in low-grade, ER-positive IDC of no specific type (IDC-NST) (82–84). Based on this, Duprez et al. suggested that PC may be a part of the ER-positive IDC-NST spectrum rather than a separate entity (58). In particular, the use of microarray-based gene expression, gene copy number profiling and RNA sequencing techniques indicated that PC is a type of luminal breast cancer with a transcriptomic profile distinct from that of graded and ER-matched IDC-NST. Furthermore, the papillary histological pattern was not due to a highly recurrently expressed fusion gene or mutation. In addition, no recurrent fusion genes supported PC. PC has fewer aberrations than IDC-NST, classified by grade and ER status at the transcriptomic level, including losses in 16q or gains in 16p and 1q (26). Analysis of 19 oncogenes showed that breast PC had gene copy pattern profiles similar to ER status and grade-matched IDC-NST, but lower p53 expression (1.6%) and fewer gene copy number abnormalities than IDC-NST (58). Minimal *p53* expression and infrequent genomic aberrations suggest that tumor cells have stable and ordinary mechanisms for self-renewal and programmed cell death. In addition, *pik3ca* mutations are observed in approximately 40% of PC, which are positive prognostic features in ER-positive IDC-NST (85, 86). Piscuoglio et al. demonstrated diverse gene expression patterns between PC and hierarchically matched IDC-NST, along with significant differences in the transcriptome profiles of EPC, SPC, and IPC (87). Genes associated with cell assembly and organization were also affected. Several genes, including laminin subunit beta 1 (*lamb1*), alpha-actinin 1 (*actn1*), and collagen were expressed at significantly lower levels in PC than in IDC-NST, in addition to genes related to cell adhesion, motility, and migration. Moreover, matrix metallopeptidase 3 (*mmp3*), matrix metallopeptidase 7 (*mmp7*), and thrombospondin (*thbs*) were expressed at significantly lower levels in PC of the breast than in IDC-NST. In contrast, genes responsible for maintaining cellular equilibrium, such as quiescin sulfhydryl oxidase 1 (*qsox1*), displayed markedly increased expression levels in breast PC compared to those in graded and ER-matched IDC-NST. Notably, *qsox1* functions as a biomarker of recurrence risk and poor survival in patients with luminal B breast cancer. Its involvement in tumor proliferation and invasion revolves around the decrease in the functional activity of matrix metallopeptidase 9 (*mmp9*) (88). Notably, EPC, SPC, and IPC demonstrated comparable genomic mutation patterns, yet their transcriptomic profiles, specifically those concerning cell migration, varied, potentially contributing to their distinct histological characteristics (58). To gain a more comprehensive understanding of these discrepancies and investigate their possible implications, additional research utilizing molecular biological techniques is crucial. In addition, exploring relevant PC high-fidelity models, such as organoids, may be an effective way to minimize the negative impact on research owing to the scarcity of this class of lesions. In 2020, Li et al. collected tumor specimens from a patient diagnosed with PC and constructed related organoids for drug sensitivity experiments. Fulvestrant showed the highest anticancer efficacy among all five tested endocrine therapeutics (89).

## Treatment and prognosis

Currently, there is no established optimal treatment strategy for IPC due to insufficient evidence-based medical research. The lack of peripheral myoepithelial cells in breast cancer generally signifies infiltration of the lesion, indicating the potential for metastasis, necessitating systemic treatment (90). In contrast, although IPC exhibits malignant histological features similar to those of IDC, the majority of IPC do not have significant invasive and metastatic capabilities. This incomplete correlation between histology and true biological behaviors is insufficient to support a systemic combination therapy approach. Therefore, we should recognize and emphasize the uncertainty in the management of this lesion and decide on systemic treatment based on surgical interventions and individual pathological features to avoid over- and undertreatment. Furthermore, in cases of mixed lesions, treatment decisions should be based on the type of lesion with the highest degree of malignancy.

Surgical treatment not only halts tumor progression, but also enhances the quality of life of patients with locally advanced disease, making it the conventional treatment for breast cancer. In order to ensure precision treatment, it is imperative that the tumor is completely removed during surgery (91, 92). The role of axillary lymph node dissection in the treatment of certain types of breast cancer with a good prognosis has been called into question. Conversely, sentinel lymph node biopsy (SLNB) is a crucial aspect of surgical management for breast cancer and is the preferred procedure for patients with clinically negative axillary lymph nodes. Although axillary lymph node metastases are uncommon in IPC, SLNB is still recommended for patients with IPC to detect any potential metastases. Moreover, breast-conserving surgery can improve the quality of life for patients after surgery and may be the best option for low-risk IPC patients. Generally, all patients who undergo breast-conserving surgery require radiotherapy. In early IPC (defined as stage T1-2 N0 disease), radiotherapy after lumpectomy is associated with improved overall survival compared with lumpectomy alone or mastectomy alone (30). When dealing with a cystic structure, it is important to consider the feasibility of flap transfer prior to surgery as IPC tends to be larger in these cases (93).

Although breast cancer presents locally, it is a systemic disease that necessitates comprehensive systemic treatment. Systemic treatment regimens are typically assigned based on the risk of breast cancer recurrence and molecular typing. Most patients with IPC are postmenopausal women with a low TNM stage, histological grading of the tumor, and molecular typing of luminal, which is a low-intermediate risk group. The efficacy of chemotherapy in this patient population lacks robust evidence and should only be considered after evaluating the results of gene expression assays and physical tolerance of elderly patients. Notably, endocrine therapy plays a critical role in the systemic treatment of Luminal breast cancer, and it has been shown to significantly enhance patient prognosis (94). IPC is mostly encountered in the elderly, and endocrine therapy is particularly suitable because of its low toxicity, efficacy, and ease of use. In 2016, a case report was published in Japan of an 83-year-old postmenopausal woman with HR+/HER2- IPC who refused surgical treatment. The patient received neoadjuvant endocrine therapy with an aromatase inhibitor, resulting in complete pathological remission of the 2 cm lesion after 12 months of treatment (95). To date, this is the only reported case of neoadjuvant endocrine therapy for IPC. This shortage of data may be attributed to the rarity of IPC, which accounts for just 0.5-1% of all breast cancer diagnoses. Further evidence-based medical studies are necessary to determine the long-term benefits of endocrine therapy in patients with IPC, owing to limited prognostic data. Furthermore, based on traditional therapeutic regimens, the active development of treatments targeting tumor-specific molecules is of great significance for the implementation of individualized breast cancer treatments. The combination of CDK4/6 inhibitors and endocrine therapy has recently become an option for treating patients with HR+/HER2- breast cancer. The use of CDK4/6 inhibitors has ameliorated the brief resistance to standard endocrine therapy, elevating breast cancer treatment to a new level of targeted combined endocrine therapy and a possible selection for IPC treatment (96).

The IPC progresses more slowly, has a lower rate of lymph node metastasis, and has a better clinical prognosis than IDC-NST (30, 34). Zheng et al. reported that patients with IPC had lower lymph node involvement than patients with IDC (11.6% vs. 32.6%), and five-year disease-specific survival (DSS) was significantly better in IPC than in IDC (97.5% vs. 93%) (26). Mitnick et al. reported a 5-year disease-free survival (DFS) rate of approximately 90% for IPC (34), and Schneider et al. reported a 10-year survival rate of 86% (31). In addition, IPC patients showed better 5-year overall survival (OS) and DFS than IDC patients matched for age, menopausal status, lymph node status, tumor size, and tumor grade. Specifically, IPC had a 92.77% OS compared to the IDC’s 87.95%, and 87.95% DFS compared to the IDC’s 80.72% (18).

Furthermore, a recent extensive retrospective study has shown that age, pathological stage, and radiation therapy are independent prognostic factors in patients with IPC. Patients who were older, had locally advanced tumor, and did not receive radiotherapy had a worse prognosis. However, the prognosis of IPC and IDC was similar, with both having comparable five-year OS rates (86.8% vs. 88.7%) (30). Similarly, Zheng et al. found that patients with IPC did not have a statistically significant survival advantage over those with IDC, even after adjusting for potential confounding factors. These inconsistent conclusions can be attributed to three reasons. First, there was no universally accepted definition of the IPC before 2003, resulting in varying interpretations among the studies, which led to disparate conclusions (97, 98). Second, papillary lesions are inherently intricate, and related studies may have had inadequate sample sizes, incorrect classifications, and insufficient elaborations (26). Finally, low lymph node metastasis, few gene copy number aberrations, low *p53* expression, and a high *pik3ca* mutation rate are thought to underlie the prognosis of IPC. Consequently, as a specific histologic type, IPC may not independently predict patient prognosis (58). An accurate prognosis necessitates further evidence-based medical research. Therefore, physicians should adopt aggressive therapeutic measures while simultaneously avoiding unnecessary treatment.

## Conclusion

Care must be taken in diagnosing IPC, as it is a rare cancer and lesions may coexist with it. This type of invasive breast cancer is most common in older women and has relatively inert biological behaviors, overtreatment should be avoided. Pathological features have been interpreted as a source of good prognosis, but genetic studies will deepen our understanding of its biological behaviors.

## Author contributions

SW: Writing – review & editing, Writing – original draft, Software, Data curation, Conceptualization. QZ: Writing – review & editing, Data curation. XM: Writing – review & editing, Writing – original draft, Visualization, Validation, Supervision, Software, Resources, Project administration, Methodology, Investigation, Funding acquisition, Formal analysis, Data curation, Conceptualization.
